# The Utah psychotropic oversight program: collaboratively addressing antipsychotic use within youth in foster care without prior authorization

**DOI:** 10.3389/fpsyt.2023.1271165

**Published:** 2023-11-03

**Authors:** Eric T. Monson, Sachi Shastri, Danli Chen, Stacy L. Madden, Brooks R. Keeshin

**Affiliations:** ^1^Department of Psychiatry, University of Utah, Salt Lake City, UT, United States; ^2^Medical Scholars Program, Augusta University/University of Georgia Medical Partnership, Athens, GA, United States; ^3^Study Design and Biostatistics Center, University of Utah, Salt Lake City, UT, United States; ^4^Department of Pediatrics, University of Utah, Salt Lake City, UT, United States

**Keywords:** youth, adolescent, foster, polypharmacy, antipsychotic, trauma

## Abstract

**Objectives:**

Fostered youth have increased risk of exposure to trauma. Antipsychotic medications are often utilized within the foster care system, potentially to address problematic behaviors that may be associated with trauma. The Utah Psychotropic Oversight Program (UPOP) was formed to support prescribers and encourage evidence-based treatment approaches for fostered youth. However, it is unclear what impact an oversight program can have on a high turnover population and without tools such as prior authorization. This study evaluates 4 years of collected data from the UPOP program for efficacy and to identify future intervention targets.

**Methods:**

Deidentified data were collected as a routine function of the oversight program over 4 years (01/2019-12/2022), from individuals aged 0–18 years old (total *N* = 8,523, 48.3% female). UPOP oversight criteria: ≤6yo + any psychotropic medication, ≥7yo + 2 or more psychotropic medications. For this analysis, youth were divided by UPOP individuals ever receiving an antipsychotic (AP) prescription (UPOP_AP; *N* = 755, 42.3% female) or not (UPOP_NAP, *N* = 1,006, 48.3% female) and non-UPOP fostered (*N* = 6,762, 48.9% female). Comparisons were made across demographic and clinical variables via ANOVA, Chi-square, unpaired *t*-test, and logistic regression.

**Results:**

UPOP_AP more likely to be older males with behavioral diagnoses, increased polypharmacy, longer duration of fostering, and higher care level. AP prescription rates dropped from 52.8 to 39.1% for males and 43.3 to 38.2% in females with unchanged number of psychotropic prescriptions and care level across 2019-2022. UPOP_AP that discontinued AP treatment had fewer average psychotropic medications, but increased antidepressant and sleep prescriptions, as compared with individuals that remained on AP.

**Conclusion:**

Youth within the foster care system receive antipsychotics at high rates and in an uneven distribution. Prescribing practices can change in the context of supportive oversight programs without components such as prior authorization, and without increasing the need for higher levels of care. Specific emphasis on the treatment of mood, anxiety, and sleep issues may also lead to greater success in discontinuing AP treatment. Oversight may support treatment providers while reducing exposure to medications with considerable side effect burden that could cause future comorbidity.

## Introduction

The foster care system plays an essential role in caring for displaced youth. Within the United States, fostering actively supports almost 400,000 youth at any given time and served over 685,000 youth within 2021 (1). Youth in foster care are at particularly high risk for prior and future exposure to traumatic experiences, with an estimated rate of >90% of these individuals being exposed to one or more traumatic events (2). Indeed, the most commonly cited reasons for requiring foster care placement include neglect, parental substance use, physical and sexual abuse, and inadequate housing conditions, all of which are direct trauma or increase risk of traumatic experiences (1). In addition, exposure to multiple traumatic experiences is more likely to result in emotional and behavioral dysregulation (3) and greater rates of mental health disorder diagnoses (4). These mental health impacts can, in fact, prolong the need for foster care and complexify individual health needs within the foster care system. The complexity of mental health needs of youth in foster care may contribute to a greater reliance on psychotropic, and particularly, antipsychotic (AP) medications to manage these symptoms within this population, recognizing that severity of emotional and behavioral dysregulation does not fully explain the increased use of such treatments within fostered youth (5).

Indeed, higher rates of polypharmacy and, in particular, utilization of AP medications within foster care youth, as compared with peers in other settings, has been consistently observed over the past several decades (6), leading to federal policy and state implemented programs designed to meet the psychiatric needs of foster care youth (7, 8). The high rate of AP use is likely multifactorial. For example, increased AP use may be related to high rates of psychiatric diagnoses and patterns of polypharmacy observed within fostered youth, noting that >40% of fostered youth seeking psychiatric care may receive medications of three or more classes, with >50% receiving prescriptions for AP medications (9). Increased diagnoses and prescriptions may arise from greater needs but may also be a symptom of systemic issues. Specifically, foster parents/caregivers may seek care for behavioral history/concerns, yet are unable to provide context or critical history from biological parents. Care is also often delivered by primary care providers that may not be trained in assessing mental health concerns in the context of foster care displacement and other traumatic experiences (10). In addition, the added strain to foster parents and risk of placement disruption in youth with challenging behaviors may increase the demand for rapid and accessible solutions, potentially also contributing to an overreliance on off-label use of AP medications (11). There may also be incentive for more severe diagnoses and complex treatment regimens to improve access to care within programs that require evidence of severe mental illness to justify reimbursement (10). Finally, access to evidence-based psychotherapeutic modalities remains limited, often with low-completion rates, even with ongoing efforts to make these interventions more accessible (12).

It is also important to consider the existing evidence base for the most effective treatments for traumatized youth. The Food and Drug Administration has not, at this time, approved any medication, AP or otherwise, for the treatment of post-traumatic stress disorder (PTSD) or other trauma-associated disorders in children. Overall, efforts to investigate medications for use in trauma-associated disorders have been limited, and multiple systematic reviews of existing studies over the past decade have not identified evidence to support the use of psychotropic medication in the treatment of PTSD for youth (13–15). Considerable support for trauma-focused psychotherapeutic modalities, such as trauma focused cognitive behavioral therapy, however, has been identified (14, 16). It is also important to note that existing literature has not formally explored how the utilization of trauma-informed psychotherapeutic approaches may or may not alter prescribing practices of psychotropic medications in community settings, though one small study identified minimal benefit in the addition of the medication sertraline to patients receiving trauma-informed psychotherapy (17). In addition, evaluations of coordinated care systems, or “wraparound” models, have shown the potential to reduce overall antipsychotic prescribing in youth with severe mental illness (18), but do not appear to reduce polypharmacy, in general, among such complex populations (19). Additional research into the potential impact of the implementation of evidence-based interventions on community prescribing practices, and particular utilization of AP medications, would be beneficial to clarify these issues.

Regardless of the cause, increased use of APs within the pediatric population is particularly concerning for multiple reasons. Fostered youth represent a significantly vulnerable population due the transitory and varied responsibilities of caregivers, including potential inconsistencies regarding identifying the individual(s) assigned to consent and administer treatments. This potential discontinuity in informed consent can impact treatment through breakdowns of communication, unclear/inconsistent roles, and variable involvement of fostered youth in shared decision-making (20). Appropriate informed consent is of particularly high importance when considering AP treatment due to the potential for long term sequelae from these medications in this population. For example, use of second-generation AP medications in youth is correlated with negative health outcomes, including increased rates of obesity, type II diabetes, and heart disease (21, 22). It has also been observed that these outcomes may be more severe within younger populations and particularly medication naïve patients (23, 24), with calls for further large scale analyses within young populations due to limited existing studies (25). In addition, the impact of antipsychotic prescription on development has not been well established (26, 27), though animal models have suggested altered function (28) and potential loss of regional cortical volumes with chronic use (29). Such findings have driven the development of statewide monitoring programs for the prescription of AP medications in youth and the study of programs designed to decrease AP use (30).

In response to these concerns, the U.S. federal government specifically released recommended monitoring guidelines for children and adolescents enrolled in Medicaid and the Children’s Health Insurance Program (CHIP) as part of the Children’s Health Insurance Program Reauthorization Act in 2009. These measures, which will be subject to mandatory reporting for children enrolled in CHIP and Medicaid nationwide starting in 2024, include the reporting of data related monitoring stimulant and AP medication prescription, along with other mental health metrics, further encouraging the development of state monitoring programs over the past decade (31). An extensive overview of state-based interventions for fostered youth was generated by Mackie et al. in 2017, noting limited publications within this area prior to this time, and electing to directly interview informants from all 50 states. A primary finding of this effort was that 88.2% of states had implemented at least one form of oversight of psychotropic prescriptions in youth, with approximately half of all programs including some form of prior authorization, requiring explicit review of recommended medication prescriptions by a panel before being approved for specific medications or classes (32). In addition, Mackie et al. prepared a systematic review of the handful of published state monitoring programs, many of which are only published as part of local government data sheets, with the review focused specifically on outcomes related to antipsychotic medications in youth, though not specific to foster care (8). This review similarly identified that the majority of published programs implemented prior authorization programs, and that this approach generally reduced antipsychotic prescription rates in youth. For example, studies on prior authorization approaches covered within the review and since implemented within Florida (33), Washington (34), and California (35) led to a decline in targeted psychotropics by 35, 49, and 56%, respectively. Two programs using an alternative approach of drug utilization review in fostered youth, only published within local government documentation, saw reported improvements in metabolic monitoring and reduced psychotropic polypharmacy, but statistical analyses of these outcomes were not available (8). Finally, one study focused on the education and consultation of providers regarding psychotropic prescribing in the state of Ohio that led to significant drops in specific population groups of both AP use and psychotropic polypharmacy, notably without a prior authorization requirement (36).

To support the delivery of safe, evidence-based and trauma-informed psychiatric care for fostered youth within the state of Utah, the Utah Psychotropic Oversight Program (UPOP) was established in 2016. This program was designed with the intention of providing oversight of prescriptions, utilizing a retrospective drug utilization review framework, within the Utah foster care system. In addition, support to prescribers was offered via a consultation line and direct provider outreach in complex polypharmacy cases to provide evidence-based and trauma-informed recommendations for treatment, including the application of trauma-informed psychosocial interventions and deprescribing guidance. In general, each foster child in Utah receives yearly mental health assessments and state contracted foster care providers are required to provide access to mental health services including psychiatric care and psychotherapy. However, similar to other parts of the country, there is a notable lack of approved mental health service providers who deliver evidence-based psychosocial interventions. Furthermore, no formal process exists within child welfare for identifying which fostered youth are receiving services beyond “individual” therapy. Therefore, creating a program that encourages appropriate utilization of evidence-based services was a high priority when creating UPOP. Finally, as has been noted, numerous states have adopted approaches that include prior authorization programs for managing AP prescriptions, but this component was not included as part of the UPOP program. The decision to not include a prior authorization component was made based on legislative support for a collaborative model, encouraging the interaction of providers to allow an opportunity to make recommendations for trauma-informed interventions, as indicated.

The State of Utah legislates the parameters of the UPOP program, including the definitions of age categories for monitored children, data variables collected, and psychotropic monitoring parameters. Foster care nurse case managers provide care coordination for all children in foster care in Utah, including the collection and entering of approved personal health data and medication regimens into a standardized system. Reports are run every quarter, identifying UPOP-qualified cases from entered data. Reports are also utilized for ongoing quarterly monitoring of qualified cases, including UPOP reviewers discussing all qualifying cases with the assigned foster care nurse case manager. When UPOP youth with five or more psychotropic medications are identified, or other complex medication regimens are identified, the reviewer will reach out to the prescriber directly via email and/or phone calls to discuss the case and provide consultation about alternatives. These communications are repeated quarterly as cases are reviewed if concerns remain. This communication allows UPOP reviewers to get additional information regarding the status of the patient, justification for AP use including current symptoms, expected monitoring parameters, previous medication trials, and discuss alternative treatments. In addition, every UPOP youth is reviewed quarterly and recommendations are provided to the child welfare team, including the case manager for the youth who is responsible for consenting to medication prescriptions on behalf of the youth and communicating with the youth’s biological parents. As such, the UPOP team, including advance practice providers and child psychiatrists, relies on collaboration and education of child welfare teams and foster care agencies, along with proactive outreach and consultation with prescribers across the state, to voluntarily carry out program recommendations. Through the efforts of collaboration, education, and consultation, UPOP attempts to effect medication change for cases that fall outside the goals of safe, evidence-based, trauma-informed care.

It is important to note that reducing rate of use of AP prescriptions alone does not necessarily indicate that best practices are being followed for the use of these medications, nor that adverse outcomes are being avoided. In addition, as noted, the effectiveness of oversight approaches that do not include the use of prior authorization has not been fully established. As such, this analysis reviews and analyzes data collected from 2019 to 2022 to better understand the outcomes associated with the program, as well as the characteristics of youth in foster care that are prescribed and deprescribed AP. Specifically, outcomes assessed include the overall rate of antipsychotic prescription, rate of fostered youth requiring high level services, average number of prescribed psychotropics, factors correlated with antipsychotic prescription, and factors correlated with successful discontinuation of antipsychotics within fostered youth. Such information may play an important role in modifying the design of monitoring programs as well as provide direction for the effective management of specific risk groups.

## Materials and methods

This study has been performed with the oversight and approval of the University of Utah Institutional Review Board. As part of ongoing oversight work, individual level data was collected monthly by UPOP staff to facilitate timely reviews. It is noted that there was a lag between program creation in 2016, initial data collection efforts beginning in 2017, and the development of a consistent data collection and reporting method, available starting 2019. Due to extensive inconsistencies and incomplete data prior to 2019, these data were not included within analyses. Therefore, for the purposes of this study, deidentified data from 2019 to 2022 were shared with the research group for processing and analysis. A total of 8,523 unique individuals, aged 0–18 years of age, were served by the Utah foster care system. Individuals entered UPOP monitoring status based on criteria determined within the legislation that created the UPOP program. Specifically, any individual ≤6yo prescribed any psychotropic medication and any individual ≥7yo prescribed 2 or more psychotropic medications was flagged for monitoring within the UPOP program. A total of 1,761 unique individuals met these criteria for UPOP monitoring, being further divided based on ever being prescribed an AP medication (UPOP_AP; N = 757) or not (UPOP_NAP; N = 1,004), throughout the reporting window.

Data were collected as part of routine monitoring by foster care nurse case managers who manually entered data provided by prescribers and foster care families into a standardized, cloud-based data management system. Note that these data do not link to electronic health records, Medicaid claims data, or pharmacy records. Therefore, specific diagnoses within fostered youth were not available, but clinical indications were collected along with prescribed medications. Data variables approved by the state for individuals flagged for inclusion in UPOP were placed within quarterly reports that were shared with the UPOP team. Data collected included basic demographic details, medications prescribed, and indications provided for prescribed medications. Medications and indications were placed within specific categories via expert consensus (Drs. Keeshin and Monson) to improve analytical power and interpretability of results. Specifically, Supplementary Table S1 describes medications that were identified as being prescribed to UPOP patients, and which were placed within mechanistic or treatment categories, as listed. Supplementary Table S2 describes listed clinical indication categories for prescribed medications, provided as justification by prescribers for the use of the medication in a given patient, divided into categories based on shared clinical characteristics as determined by expert consensus. These defined categories were used as variables within study analyses. Data collected for non-UPOP individuals was limited to basic demographic information, including age, sex, ethnicity, region, placement level, and placement reason. It is noted that placement level was a variable available for all subjects, providing insight into the complexity of care required for a given individual; increased levels of specialization and care are represented by each increase in level. For evaluation purposes in this study, level of care was broken into two categories, low and high. Placement levels 1–3 represent foster family placement, classified as lower placement specialization requirements, and levels 4–7 plus individualized residential treatment services (IRTS) represent group home, residential, and more specialized inpatient treatment environments indicating a higher placement requirement.

Overall demographic variables were assessed across all UPOP and non-UPOP fostered youth utilizing chi-square, unpaired *t*-test, and ANOVA comparisons, as appropriate for the variable assessed. Demographic and clinical factors were also examined year-by-year to understand prescription and population trends throughout the course of the monitoring window. Primary study analyses were then conducted to compare factors contributing to antipsychotic use. Individuals were grouped based on antipsychotic prescription patterns. Specifically, UPOP_AP were compared with UPOP_NAP across demographic factors, indications, and prescribed medications. A secondary analysis was performed to examine UPOP_AP individuals who were observed to have the AP discontinued versus UPOP_AP who remained consistently on antipsychotic medication. The purpose of this secondary analysis was to better clarify factors that predicted discontinuation of AP use. Primary and secondary analyses utilized logistic regression modeling, performed with the inclusion of covariates considered to potentially bias results, including sex, ethnicity, age, and months in custody. Primary and secondary analyses were also corrected for multiple comparisons via the Benjamini-Hochberg method with a false discovery rate threshold of 0.05.

## Results

Demographic variables compared across groups, UPOP_AP, UPOP_NAP, and all other fostered youth are provided in Table 1. Clinical variable comparisons, including categorized prescribed medications and indications provided for prescriptions are provided in Supplementary Table S3. Among these comparisons, there were several distinguishing differences for UPOP_AP as compared with the other fostered groups. UPOP_AP are, on average, older, with an average age of 13.9 yo when entering foster care as compared with 10.4 yo, and 5.9yo for UPOP_NAP and non-UPOP fostered youth, respectively. UPOP_AP are more likely to be male (58%) as compared with UPOP_NAP (52%) and non-UPOP fostered youth (51%). Finally, UPOP_AP spend more time, on average, within foster care with 36.6 months vs. 25.8 and 14.5 months for UPOP_AP vs. UPOP_NAP and non-UPOP, respectively, and require a higher level of care within the foster system, with 88% requiring level 4 and above foster care services as compared with 59% of UPOP_NAP and 20% of non-UPOP fostered youth.

**Table 1:** Demographic characteristics of UPOP and non-UPOP fostered youth.

Characteristic	UPOP_AP	UPOP_NAP	Non-UPOP	*p*
*Age at first observation*				
≤ 6yo	21 (3%)	296 (29%)	4,053 (60%)	< 0.001
7yo to ≤12yo	178 (24%)	258 (26%)	1,670 (25%)	
≥ 13yo	556 (74%)	452 (45%)	1,039 (15%)	
*Sex*				
F	319 (42%)	486 (48%)	3,309 (49%)	< 0.01
M	436 (58%)	520 (52%)	3,453 (51%)	
*Reported race*				
American Indian Alaska Native	26 (3%)	25 (2%)	215 (3%)	< 0.001
Asian	2 (0%)	8 (1%)	32 (0%)	
Black African American	43 (6%)	55 (5%)	257 (4%)	
Multi Racial Other Race Unknown	7 (1%)	17 (2%)	128 (2%)	
Native Hawaiian Other Pacific	5 (1%)	11 (1%)	118 (2%)	
Unknown	2 (0%)	5 (0%)	122 (2%)	
White	670 (89%)	885 (88%)	5,890 (87%)	
*Reported ethnicity*				
Hispanic	142 (19%)	201 (20%)	1,650 (24%)	< 0.001
Non-hispanic	605 (80%)	792 (79%)	4,814 (71%)	
Unable to determine	7 (1%)	12 (1%)	249 (4%)	
Unknown	1 (0%)	1 (0%)	49 (1%)	
*Utah region*				
Eastern	60 (8%)	81 (8%)	543 (8%)	< 0.01
Northern	150 (20%)	253 (25%)	1737 (26%)	
Salt Lake Valley	252 (33%)	360 (36%)	2,300 (34%)	
Southwest	85 (11%)	108 (11%)	675 (10%)	
Western	208 (28%)	204 (20%)	1,507 (22%)	
*Foster care level*				
Fostered care level ≤ 3	88 (12%)	414 (41%)	5,372 (80%)	< 0.001
Fostered care level ≥ 4	665 (88%)	591 (59%)	1,328 (20%)	
Average months in custody	22.2	18.7	11.2	< 0.001
*Reason for fostered placement*				
Parental relinquishment, abandonment, adoption failure	347 (46%)	305 (30%)	1,671 (25%)	< 0.001
Ungovernable, delinquent, status offense	106 (14%)	60 (6%)	92 (1%)	< 0.001
Neglect	227 (30%)	512 (51%)	4,028 (60%)	< 0.001
Physical abuse	51 (7%)	108 (11%)	838 (12%)	< 0.001
Sexual abuse	24 (3%)	22 (2%)	130 (2%)	N.S.
*Psychotropic medications*				
Maximum psychotropic Rx	2.9	1.5	-	< 0.001

Rx, prescription; Reported percentages are within respective groups; medication data is not available for non-UPOP fostered youth.

**Figure 1:**
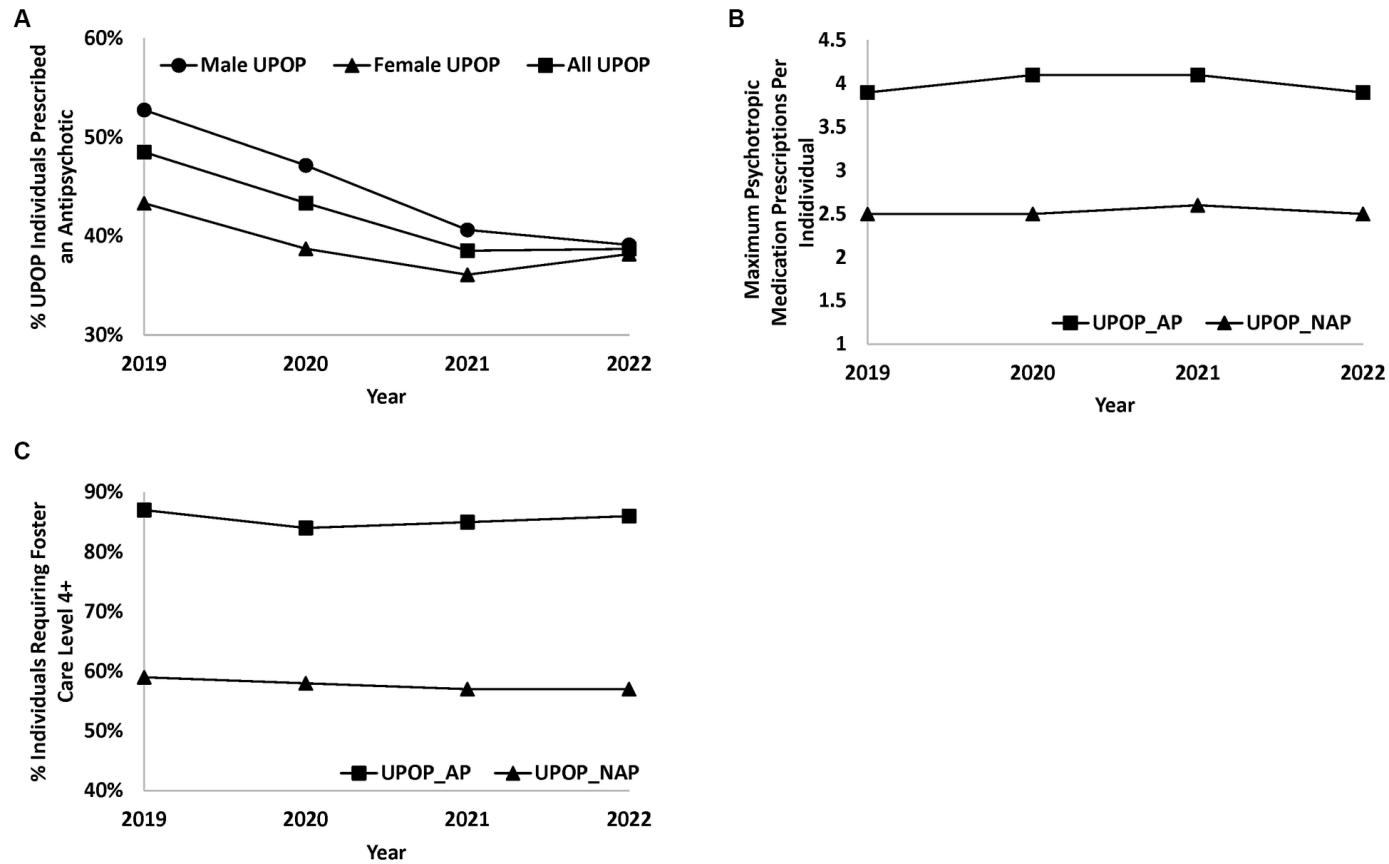
Trends in medication prescription rates and required level of care for UPOP youth. Line plots demonstrating various trends in medication and level of care requirements in UPOP youth. **(A)** Represents the percentage rate of antipsychotic prescription across male, female, and all UPOP youth from 2019 and 2022. **(B)** Represents the average count of the maximum concurrent psychotropic prescriptions per individual youth within the UPOP_AP and UPOP_NAP groups. **(C)** Demonstrates percentage rate of UPOP youth requiring foster care level 4 or above services.

Year-by-year demographic details and information on the UPOP_AP, UPOP_NAP, and non-UPOP fostered individuals are represented within Supplementary Tables S4–S6, respectively. Several significant trends were appreciated throughout this time frame, and key trends are demonstrated in Figure 1. Overall AP prescriptions were observed to decrease from 2019 to 2022, dropping from 48.5% of all UPOP youth to 38.7%. These reductions were more pronounced in males as compared with females in UPOP youth, with totals in males dropping from 52.8 to 39.1% as compared to females dropping from 43.3 to 38.2% from 2019 to 2022. Despite these decreases in AP use, fostered youth requiring higher levels of care remained relatively stable from 2019–2022 across all groups, with 84–87%, 57–59%, and 16–20% requiring more specialized care than foster family placement for UPOP_AP, UPOP_NAP, and non-UPOP youth, respectively. Average psychotropic medication count was also observed to remain stable despite the reduction in AP prescription throughout the observation window, with UPOP_AP youth consistently receiving more medications (3.1–3.3 average prescribed psychotropics) than UPOP_NAP youth (1.9–2.0 average prescribed psychotropics). Similarly, average time in foster care remained stable across the 4 years, with consistently higher averages for UPOP_AP youth (32.9–35.1 months in custody) as compared with UPOP_NAP (23.9–26.5 months in custody) and non-UPOP youth (13.0–14.0 months in custody).

Primary analyses were focused on the identification of factors associated with AP prescription, comparing UPOP_AP versus UPOP_NAP. Significant results from these comparisons are presented in Table 2, with all results available for review in Supplementary Table S7. These analyses demonstrated that factors most significantly associated with AP prescription included being prescribed more psychotropic medications (OR = 2.4, 95%CI = 2.2–2.7), having a listed indication for medications of a mood disorder (OR = 7.3, 95%CI = 5.3–10.0), and requiring a higher level of care than that available from foster family placement (foster care level IV and above; OR = 3.2, 95%CI = 2.4–4.2). Specific medication classes were also seen more frequently in the UPOP_AP youth, including alpha modulators (alpha 2 agonists and alpha 1 antagonists; OR = 2.4, 95%CI = 1.9–3.0), antiepileptic/mood stabilizers (OR = 2.6, 95%CI = 2.0–3.4), and laxative/bowel regimen medications (OR = 2.4, 95%CI = 1.8–3.2). Additional prescription indications more frequently seen in UPOP_AP included gastrointestinal disorders (OR = 2.3, 95%CI 1.8–2.9), trauma-associated disorders (PTSD, adjustment, and attachment disorders; OR = 2.9, 95%CI = 2.0–4.2), and behavioral disorders (attention, conduct, and oppositional defiant disorders as well as “behavioral problems”; OR = 1.9, 95%CI = 1.5–2.7). Finally, UPOP_AP differed in the primary reason they were placed within foster care. Specifically, UPOP_AP were less likely to have a primary placement reason of “neglect” than their peers (OR = 0.51, 95%CI = 0.41–0.64) and more likely to have a primary placement reason of “parent condition” or “ungovernable” (ORs = 1.7 and 1.6, 95%CI = 1.3–2.1 and 1.2–2.3, respectively).

**Table 2:** UPOP_AP vs. UPOP_NAP significant results.

Type	Variable	OR	ORL95CI	ORU95CI	Corrected *p*
Demographic	Maximum psychotropic Rx count	2.40	2.16	2.67	1.69E-57
Rx Indication	Mood disorder	7.27	5.28	9.99	4.67E-33
Demographic	Fostered care level	3.17	2.41	4.18	3.04E-15
Drug	Alpha down	2.39	1.93	2.97	2.85E-14
Drug	Cation channel blocker	2.64	2.04	3.41	9.04E-13
Rx Indication	GI	2.29	1.80	2.91	1.12E-10
Demographic	Neglect	0.51	0.41	0.64	1.62E-08
Drug	Laxative	2.39	1.79	3.20	2.53E-08
Rx Indication	Trauma	2.92	2.04	4.16	2.33E-08
Rx Indication	Behavioral disorder	1.90	1.51	2.38	2.65E-07
Rx Indication	Metabolic disorder	2.07	1.59	2.69	2.88E-07
Rx Indication	Anxiety	1.75	1.41	2.17	2.34E-06
Drug	Other drug	1.79	1.43	2.26	2.71E-06
Rx Indication	Other diagnosis	2.16	1.59	2.94	3.34E-06
Rx Indication	Skin	2.19	1.60	2.99	4.12E-06
Drug	Supplement	1.77	1.39	2.26	1.53E-05
Demographic	Parental relinq/abandon/failure	1.66	1.34	2.06	1.46E-05
Rx Indication	Other psychiatric diagnosis	5.58	2.49	12.48	1.01E-04
Drug	PPI	2.18	1.50	3.17	1.52E-04
Drug	Atypical antidepressant	1.58	1.27	1.97	1.48E-04
Drug	Antidiabetic	3.27	1.84	5.80	1.57E-04
Drug	Steroid	1.88	1.38	2.56	1.70E-04
Drug	Acne	2.19	1.47	3.28	3.61E-04
Rx Indication	Developmental	8.17	2.48	26.91	1.52E-03
Drug	Dopamine up	4.66	1.94	11.18	1.50E-03
Rx Indication	Pain	1.64	1.24	2.19	1.59E-03
Rx Indication	Infection	1.60	1.19	2.16	5.21E-03
Drug	Antipyretic	1.61	1.18	2.20	6.68E-03
Drug	Antiemetic	2.30	1.32	4.04	8.05E-03
Drug	SNRI	1.77	1.19	2.65	1.18E-02
Rx Indication	Allergy	1.44	1.11	1.85	1.20E-02
Demographic	Youth Ungov/Delinq/StatOff	1.63	1.15	2.31	1.21E-02
Drug	Lithium	4.58	1.54	13.58	1.26E-02
Demographic	Average months in treatment	1.01	1.00	1.02	1.97E-02
Drug	Non-opioid analgesic	2.86	1.27	6.43	2.18E-02
Drug	Antibiotic	1.47	1.09	1.98	2.14E-02
Rx Indication	Respiratory	1.34	1.04	1.71	3.96E-02

OR, odds ratio; L/U95CI, lower and upper 95% confidence interval; GI, gastrointestinal; PPI, proton pump inhibitor; SNRI, selective serotonin reuptake inhibitor; Relinq, relinquishment; Ungov, ungovernable; Delinq, delinquent; StatOff, status offense. For specific definitions of each categorical variable, refer to Supplementary Table S1 for all “Drug” type variables, and Supplementary Table S2 for all “Rx Indication” type variables.

Secondary analyses were focused on the identification of factors associated with discontinuation of antipsychotics within UPOP_AP youth. Specifically, UPOP_AP that were observed to discontinue AP medications at any time during their care were compared with UPOP_AP who remained on AP medications throughout their care. Significant results for these analyses are reported in Table 3, with all results available for review in Supplementary Table S8. These analyses identified that individuals who discontinued APs tended to be in foster care longer than those that remained on APs (OR = 1.08, 95%CI = 1.06–1.09). In addition, UPOP_AP youth who discontinued AP treatment were, on average, taking fewer psychotropic medications (OR = 0.57, 95%CI = 0.50–0.65), and were more likely to be taking an SSRI or other antidepressant medication (ORs = 1.9 and 1.9, 95%CI = 1.4–2.6 and 1.4–2.5 for SSRI and atypical antidepressant classes, respectively). Finally, UPOP_AP youth that discontinued AP treatment were also more likely to have a sleep disorder listed as an indication for medication administration and be prescribed melatonin (ORs = 1.8 and 1.7, 95%CI = 1.2–2.6 and 1.2–2.4, for sleep indications and melatonin prescription, respectively).

**Table 3:** UPOP_AP that discontinued AP vs. UPOP_AP consistently on AP.

Type	Variable	OR	ORL95CI	ORU95CI	Corrected *p*
Demographic	Average months in treatment	1.08	1.06	1.10	1.25E-21
Demographic	Average psychotropic Rx	0.57	0.50	0.65	7.91E-16
Drug	SSRI	1.93	1.41	2.64	7.25E-04
Drug	Atypical antidepressant	1.86	1.36	2.53	1.28E-03
Rx Indication	Sleep	1.80	1.27	2.55	1.07E-02
Drug	Melatonin	1.73	1.25	2.39	1.02E-02
Drug	Antibiotic	1.75	1.18	2.60	4.90E-02

Rx, prescription; SSRI, selective serotonin reuptake inhibitor. For specific definitions of each categorical variable, refer to Supplementary Table S1 for all “Drug” type variables, and Supplementary Table S2 for all “Rx Indication” type variables.

## Discussion

At a population level, exposure to AP medications among foster care youth within the UPOP program is quite high and decreased over the 4-year observation window. Factors associated with AP prescription included males, older individuals, and longer duration of foster care. This finding falls in line with prior studies evaluating prescription patterns in youth, showing that adolescent males are more likely to be prescribed APs (37) and have greater rates of psychotropic polypharmacy (38). The population level decrease in AP prescribing over time was most notable among males and those with a primary reason provided for foster care placement of behavioral challenges, listed as “ungovernable.” It is important to recognize that even with the decrease, the use of antipsychotics among foster care youth remains high. Similar patterns have been seen within other state programs, with a gradual drop of AP use over years of implemented programs (33–36), with likely similar, but unreported, trends across the country as individual state programs have been implemented in the majority of states at this time (8, 32). In addition, reported programs similarly noted ongoing elevated rates of AP use in these populations as compared with peers despite the implemented programs. It is suspected that similar challenges are contributing to elevated AP use within the state of Utah as those observed within other regions, as described within the introduction, including ongoing limited access to evidence-based psychosocial interventions and other systemic issues. Importantly, those affected by increased foster placement as well as elevated psychotropic and antipsychotic use were not demographically representative of the state. Specifically, it was noted that African-American children made up 4% of total fostered youth and 6% of the UPOP_AP group, despite African-Americans representing only 1.6% of the Utah state population from 2020 census data (39). Alternatively, individuals reporting Asian descent made up less than 1% of any fostered group, despite census data reporting this population representing 2.8% of the state population in 2020. Other racial groups were represented at relatively expected levels based on reported state demographics (39). Despite these complexities, this is the first peer-reviewed publication to demonstrate overall decreased use of antipsychotics associated with an oversight program focused on a drug utilization review intervention and that does not include a prior authorization component. Rather, continuous monitoring, education of child welfare staff, foster care agencies, and proactive outreach and consultation with prescribers across the state were the primary tools available to the oversight program.

Whether they arrived in foster care on antipsychotics or were initiated on antipsychotics while in foster care, 49.8% of youth in foster care treated with antipsychotics continued that treatment for the duration of the study period or until leaving foster care. Interestingly, most youth prescribed an antipsychotic did not have a listed indication of an identified primary psychotic disorder or bipolar disorder. Rather, it was noted that antipsychotic medications were most frequently prescribed for the indications of “mood disorder” and “depression,” together representing 64.5% of listed indications for all prescribed antipsychotic medications. “Behavioral Problems” (rather than a specific diagnosis) was the third most cited indication for antipsychotic prescription, making up 8.8% of listed indications. Importantly, FDA approved indications for antipsychotic use in child and adolescent populations include schizophrenia, bipolar disorder, irritability in autism, and Tourette’s syndrome (40). All of the listed approved indications for AP prescription in children and adolescents, combined, represented only 4.3% of listed indications within the collected data. These findings are similar to prior observations within nationwide use of antipsychotics in youth, with considerable trends toward off-label use for listed indications of mood and behavioral symptoms (37).

A factor hypothesized to be correlated with increased reliance on antipsychotic use among youth in foster care is the lack of evidence-based trauma-informed resources. It is well established that less densely populated regions are more likely to lack specialized mental health resources for youth, and Utah is no exception. In fact, a state such as Utah, with its heterogenous geographic distribution is an ideal setting to evaluate the impact of rurality on treatment practices. Importantly, region of the state did not appear to have a substantial impact on either AP prescription patterns or ongoing AP use among youth in foster care. More importantly, prescribing practices appeared to play a significant role in predicting whether youth would be removed from antipsychotics throughout their treatment. Specifically, prescription of antidepressant medications and treatment of sleep issues were correlated with greater rates of discontinuing antipsychotic medications. The role of sleep dysregulation, particularly, following trauma exposure is well established as a critical therapeutic target that, if not addressed, may worsen pathology, including behavioral disturbances (41, 42) and development of trauma-associated symptoms (42, 43). Antidepressants, on the other hand, have not been clearly demonstrated to effectively manage PTSD symptoms in children (13), but can be effective in treating underlying anxiety and depression, which when present, can drive reactive behaviors. More research is needed to better understand the role and impact of targeting sleep, anxiety, and/or depression among youth in foster care and how these interventions may impact the use of AP medications within this population.

Age is also an important factor associated with AP presence and persistence among youth in foster care, and which was clearly observed within the presented data. Older youth have more opportunity for the accumulation of trauma and other adversities, which, if present, increase the risk for traumatic stress and potential for emotional/behavioral reactivity. In addition, older youth are also more likely to enter into foster care with established emotional/behavioral challenges than younger youth. Similarly, adolescents represented the single largest groups receiving AP medications in efforts to evaluate nationwide prescribing trends (37). However, given that AP prescription was also associated with time in foster care, clear opportunities appear to exist to educate providers and implement preventative and early intervention strategies and may speak to the bidirectional association between time in foster care and AP use where multiple placements increased risk of AP use among fostered youth (44). Such measures may decrease the need/risk for AP use in children, especially those that may experience foster care placements that last months to years. This is particularly demonstrated by the additional association within our data of youth that spent longer in foster care, on average, also had a greater likelihood, or perhaps opportunity, to have their antipsychotic medication discontinued with the consultative support of the UPOP program.

In our data, AP use also appears to be an appropriate proxy for psychiatric treatment complexity. There was a high level of association between those youth who were on an antipsychotic and the number of psychiatric medications prescribed to youth receiving oversight. However, it should be noted that, although antipsychotic prescriptions decreased significantly over the course of the study, there was no clear change observed in the average number of psychiatric medications prescribed to youth in foster care receiving oversight. This apparent initial association, but possible decoupling with AP changes over time is worth further exploration. It could be that a result of the program is to move prescribers to medications with less risky side effect profiles, yet those youth who continue to have significant emotional/behavioral concerns and/or are difficult to manage are still receiving significant psychotropic polypharmacy. Of particular importance, it has been previously observed in the literature that a reduction in the rate of prescribing antipsychotics may, inadvertently, increase the likelihood of certain affected individuals to require a higher level of care (45), though a pattern of movement toward higher levels of care was not observed within the presented data.

The lack of an overall drop in use of psychotropic medications despite reduced AP could potentially be related to the lack of availability/access of comprehensive, evidence based psychosocial programs to better address trauma symptoms during the course of the study. However, as limited evidence currently exists as to the potential to reduce polypharmacy within youth with serious mental health issues with coordinated care interventions (19), more research into the impact of community implementation of specific, evidence-based modalities is needed. Thus, the changes over time, while encouraging, are only a small step towards improving overall care for foster care youth with complex mental health needs.

## Limitations

It is noted that this study had several limitations. Among these were substantial limitations in the data collected and subsequent analyses that could be completed. For example, though basic data were collected on important health metrics such as weight and BMI, there was very limited consistency in the collection of these data, leading to an inability to reliably or effectively evaluate these variables for evidence of adverse outcomes. In addition, laboratory values were not routinely available as part of the data due to limitations in the collection and approval process for allowed data. Finally, data collection did not include access to electronic health record data including specific diagnoses from international classification of diseases coding and clinical encounter information which could provide considerable insight into the severity of illness and acuity of specific patient subgroups. The lack of specific diagnoses also limits the interpretability of the appropriateness of AP prescription as only the listed indication for the prescription was available for consideration within this study. Such inconsistencies and incomplete records point to systematic challenges with the creation and maintenance of a monitoring program and provide opportunities for improvement in future iterations of the program.

In addition, the data available for evaluation was limited to biological male and female gender assignments only, even though there are a number of different genders represented in the Utah Foster Care system, and so we recognize that our analysis when it pertains to gender is incomplete. However, it is still notable that youth identified as male were significantly more likely to be in both the AP (58% male) and persistent AP (62% male) categories. This is consistent with various other reports (9, 46) and likely reflects that the reasons for AP use are not homogenous across genders. Therefore, efforts to limit AP use among different populations may benefit from including gender or other associated factors that may drive AP prescribing practices.

Unfortunately, data were also not available regarding the availability and utilization of evidence-based psychosocial interventions. In addition, the small numbers of individuals within specific subgroups of this data, such as those moving between higher levels of care, as well as the limited timeframe of this study limited power to appropriately assess such trends. Other limitations for this project include relying on data regarding medications entered into a child welfare cloud-based system, potentially leading to a delay between medication changes and those changes being reflected within the collected data.

## Future directions

Future analyses would benefit from more detailed examinations of how patterns of treatment change over time, particularly monitoring shifts in levels of care and specific changes in medication regimens, to better understand possible benefits and adverse events. Analyses evaluating the systematic influence of prescriber level, prescribers from specific regions, or other possible clusters may also be of potential benefit, particularly as such clusters of prescribers have been previously observed elsewhere (47), and may help identify specific aspects of culture or training that could be addressed within the program. In addition, findings from this effort suggest that further investigation may be warranted of the potential for reducing AP need within fostered youth through focused symptom management of sleep, anxiety, mood, and possibly other areas.

In addition, future efforts would also be considerably benefited through the collection of other key data, including information from electronic health records such as diagnoses and episodes of clinical care, and laboratory values for typical monitoring parameters of AP and other medications requiring regular follow-up. Such information would allow far more detailed evaluation of patient acuity, comorbidity, and side-effect burden within specific patient populations, as well as allow better evaluation of potential benefits and unintended consequences of reductions in AP use. Finally, implementing systematic detailed tracking of interventions youth are receiving, such as TF-CBT, would also allow more detailed analyses as to how patterns of access to evidence-based treatments are shifting over time and subsequent correlated changes, if any, within prescribing practices.

## Conclusion

The presented data represents supportive evidence for potential benefits of an oversight program in reducing AP burden in fostered youth absent a prior authorization requirement, a relatively understudied area. Moreover, these interventions did not appear to lead to increased burden on higher levels of foster care and may have encouraged the targeting of other symptoms with alternative, potentially safer, medication profiles. At this time, even with the discussed interventions, AP and psychotropic use remains high in fostered youth. Further efforts to track access toalternative evidence-based interventions in conjunction with patterns of AP and other psychotropic medication use could be highly valuable for guiding future oversight and treatment efforts within fostered youth.

## Data availability statement

The original contributions presented in the study are included in the article/Supplementary material, further inquiries can be directed to the corresponding author.

## Ethics statement

The studies involving humans were approved by University of Utah Institutional Review Board (IRB). The studies were conducted in accordance with the local legislation and institutional requirements. Written informed consent for participation was not required from the participants or the participants’ legal guardians/next of kin in accordance with the national legislation and institutional requirements.

## Author contributions

EM: Conceptualization, Data curation, Formal analysis, Investigation, Methodology, Supervision, Visualization, Writing – original draft, Writing – review & editing. SS: Data curation, Investigation, Writing – original draft, Writing – review & editing. DC: Data curation, Formal analysis, Investigation, Methodology, Validation, Writing – review & editing. SM: Conceptualization, Project administration, Supervision, Writing – review & editing. BK: Conceptualization, Investigation, Methodology, Project administration, Resources, Supervision, Writing – original draft, Writing – review & editing.
